# Obesity treatment across healthcare levels: collaboration and digital solutions - a qualitative study of health professional and patient perspectives

**DOI:** 10.1186/s12913-026-14155-4

**Published:** 2026-02-06

**Authors:** Ingrid Sørdal Følling, Marie Waage Eikil, Rønnaug Ødegård

**Affiliations:** 1 Department of Clinical and Molecular Medicine, Faculty of Medicine and Health Sciences, Norwegian University of Science and Technology, Trondheim, Norway; 2 Centre for Obesity Research and Innovation, Clinic of Surgery, St. Olavs Hospital, Trondheim University Hospital, Trondheim, Norway

**Keywords:** Obesity treatment, Digital solutions, Collaboration, Patient perspectives, Health professional perspectives, Primary healthcare, Specialist healthcare, Healthcare levels

## Abstract

**Background:**

Obesity is a chronic condition, and long-term weight loss through lifestyle treatment remains challenging. Although early lifestyle interventions and coordinated care across healthcare levels are widely recognized as important, referral pathways and follow-up support often remain unclear for both patients and health professionals. Digital solutions may improve communication, knowledge sharing, and continuity of care; however, most tools target individual lifestyle change and do not support collaboration across healthcare levels. This study aimed to explore health professional and patient perspectives on how collaboration and digital solutions can improve obesity care across healthcare levels.

**Methods:**

A qualitative design using triangulation of data sources with focus group interviews with health professionals (*n* = 12 across two focus groups) and individual interviews with patients (*n* = 12) was employed. Various health professionals from primary and specialist care, working with patients with obesity, were recruited via snowball sampling, whereby some professionals recommended colleagues with relevant experience. Patients were purposeful recruited for their experience with obesity treatment both in primary and specialist care. Both focus group and individual interviews followed semi-structured interview guides addressing experiences with obesity treatment, patient–professional relationships, collaboration across healthcare levels, and the use of digital solutions. All interviews were audio-recorded and analysed using thematic analysis.

**Results:**

Analysis of the interviews revealed four key themes: (1) Communication, trust, and experiences of stigma in patient–professional interactions; (2) Knowledge gaps and coordination across primary and specialist healthcare; (3) Interdisciplinary collaboration in obesity care pathways; (4) Experiences with digital solutions and in-person care in obesity treatment.

**Conclusions:**

Successful implementation of digital solutions requires alignment with patient needs while preserving the therapeutic relationship through in-person care. Patients were generally receptive to increased collaboration and shared responsibilities, whereas health professionals expressed more caution. Strengthening collaboration across healthcare levels, combined with thoughtful use of digital solutions, may improve patient-centered obesity treatment.

**Supplementary Information:**

The online version contains supplementary material available at 10.1186/s12913-026-14155-4.

## Background

Obesity is a progressive, chronic, and relapsing disease that significantly increases the risk of comorbidities [1]. Over the past 40 years, the global prevalence of overweight and obesity has risen substantially [2], and in Norway, 73% of the population is either overweight or obese [3]. Lifestyle interventions, including dietary changes, physical activity, and behavioral support, are recommended as first-line treatment for obesity in both international [4, 5] and Norwegian clinical guidelines [6]. However, the implementation and uptake of these interventions in routine clinical practice vary across healthcare settings.

Achieving and maintaining long-term weight loss is challenging for many people with obesity [7]. Early lifestyle interventions are therefore considered essential for sustained weight management and reducing obesity-related comorbidities [8]. In Norway, national guidelines emphasize the central role of primary healthcare in obesity treatment [6]. Patients with a body mass index (BMI) ≥ 35 kg/m² with comorbidities or ≥ 40 kg/m² may be referred to specialist healthcare services [9]. However, referral pathways and post-treatment support are often unclear to both patients and health professionals. This lack of structure underscores the need for improved collaboration across healthcare levels and provides a rationale for exploring digital solutions as a means to support coordination and continuity of care [10]. The use of digital solutions and solutions accelerated during the COVID-19 pandemic, including within obesity care [11]. Integrating digital solutions into healthcare provides an opportunity to support lifestyle change [12], potentially improving weight-loss outcomes through increased accessibility [13, 14]. However, most digital solutions cannot replace patient–professional relationships, and in-person lifestyle programs remain the recommended approach when feasible [15]. While some patients and health professionals find digital solutions useful, experiences indicate they cannot fully substitute face-to-face encounters due to individual needs and preferences [16]. People with obesity often experience stigma, making the patient–professional relationship crucial for motivation, adherence, and long-term success [17]. Addressing difficult emotions, such as shame, can further help reduce stigma [18]., as perceived stigma has been linked to lower trust and poorer outcomes in primary car [19]. Digital interventions can also provide psychological and behavioral benefits [20], supporting communication, patient empowerment, and self-management [21]. When integrated with health system resources, such interventions may complement personalized care and promote clinically meaningful weight loss [22]. Most apps target individual lifestyle change and do not support collaboration across healthcare levels [23]. Nonetheless, digital solutions have potential to enable early, individualized interventions and ensure continuity when patients transition between primary and specialist care. While international studies on digital solutions in obesity care are abundant, little is known about how health professionals and patients in Norway perceive these tools relative to in-person care, particularly regarding collaboration across healthcare levels. To address this gap, this study aimed to explore how patients and health professionals perceive the role of digital solutions and in-person care in supporting collaboration and patient-centered obesity treatment in Norway.

## Methods

We applied a qualitative design using triangulation of data sources, combining focus group interviews with health professionals and individual semi-structured interviews with patients.

Two focus group interviews with health professionals were conducted digitally via Microsoft Teams. The first focus group took place in September 2021 and the second in December 2021, lasting 128 and 122 min, respectively. The first author (ISF) facilitated the interviews, while the last author (RØ) observed. Health professionals were recruited through snowball sampling. Initially, three specialists in obesity treatment working in secondary care were invited by the research team and asked to recommend colleagues from primary care. In total, seven health professionals were recruited and agreed to participate in the first focus group interview, while five attended the second focus group interview (*n* = 12 across the two focus groups). Health personnels´ ages ranged from 28 to 61 years. Their characteristics are presented in Table 1.

**Table 1:** Health personnel characteristics (*n* = 12)

Characteristics	*N* (12)
**Gender**	
Female	9
Male	3
**Occupation**	
General Practitioner	3
Physician in Obesity Clinic	3
Physiotherapist	2
Clinical nutritionist	2
Nurse	2
**Health Care level**	
Primary Healthcare	6
Specialist Healthcare	6
**Clinical experience**	
< 5 years	2
5–10 years	4
15–20 years	6

Individual semi-structured interviews with patient were conducted individually via Microsoft Teams between November 2023 and January 2024. All interviews were audio recorded and lasted on average 44 min (range 26–62 min). The second author (MWE) conducted the interviews. Patients were recruited using a strategic sampling approach. The research team contacted Healthy Life Centres in primary healthcare across Central Norway, provided information about the study, and asked staff to invite patients who met predefined inclusion criteria: BMI ≥ 30 kg/m² (obesity class I or higher) and prior experience with obesity treatment in both primary and specialist healthcare. This approach aimed to include participants with relevant experiences and a range of ages and genders. Altogether, 15 patients were invited, of whom 12 (10 women) agreed to participate. Patients’ ages ranged from 19 to 73 years (see Table 2).

Prior to participation, patients were informed about the interviewers’ roles and professional background, and all provided informed consent.

**Table 2:** Patient characteristics (*n* = 12)

Characteristics	*N* (12)
**Gender**	
Female	10
Male	2
**Age**	
18–44 years	4
45–54 years	3
55–75 years	5
**Body Mass Index (BMI)**	
< 40	4
40 - < 45	7
≥ 50	1
**Years in Obesity Treatment**	
< 3 years	3
5–10 years	2
15–20 years	3
> 20 years	4

The research team consisted of three female researchers (ISF, MWE, and RØ). ISF holds a PhD and has more than 15 years of experience with qualitative research. MWE is a registered nurse with clinical experience and training in qualitative interviewing as part of her MSc degree. RØ is a medical doctor with a PhD and serves as the leader of the Obesity Research Centre in Mid‑Norway; she has supervised qualitative studies at both the master’s and PhD levels. None of the interviewers had any prior personal relationship with participants. Prior to the interviews, participants were informed about the interviewers’ professional backgrounds and the purpose of the study. Potential interviewer bias related to experience, professional roles, and prior research engagements was addressed through ongoing reflexive discussions within the research team throughout the study.

**Fig. 1:**
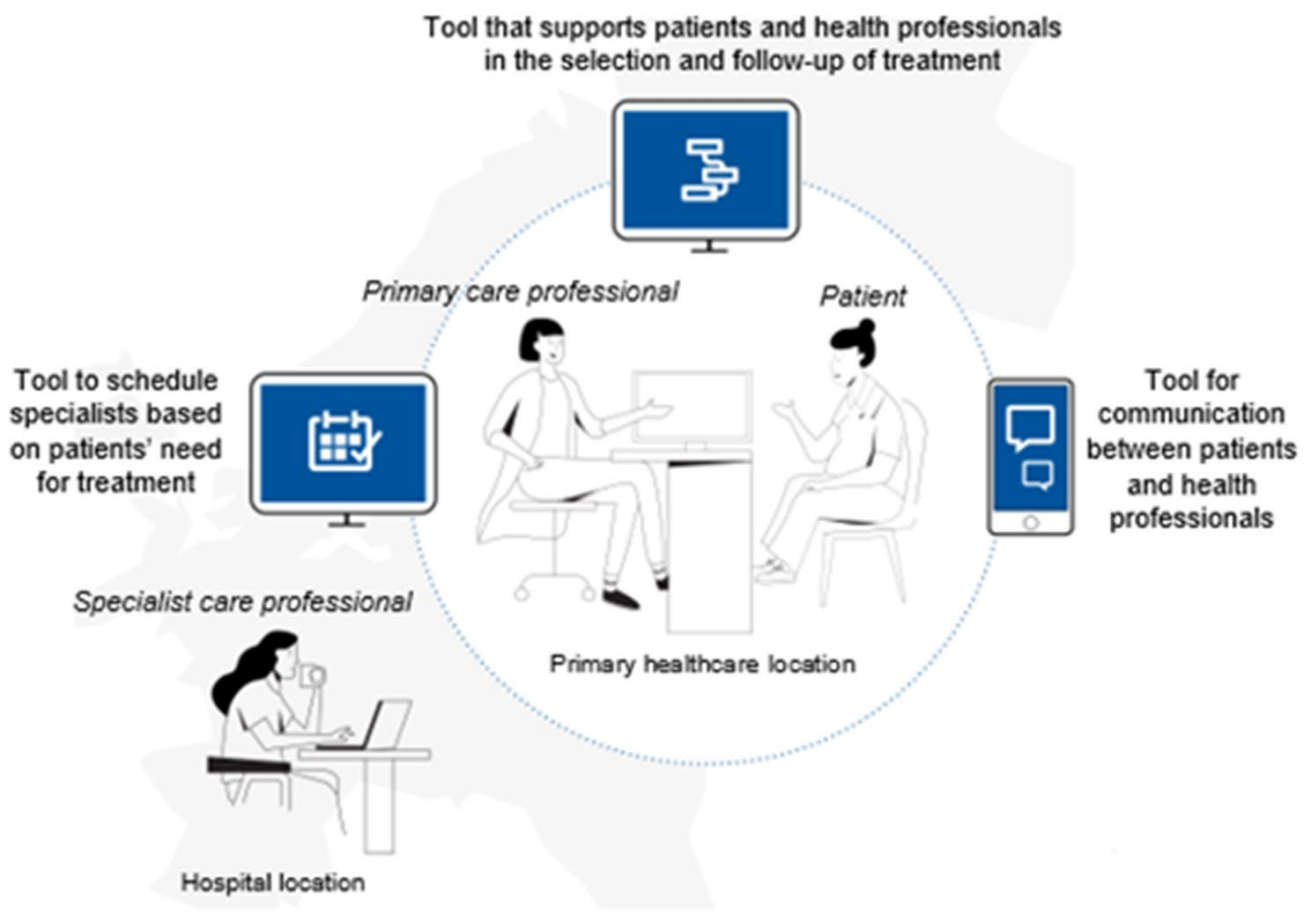
Digital solutions to support communication and coordination in obesity care across healthcare levels. Figure legend: This illustration shows how digital solutions can facilitate communication and collaborative care between patients, primary care professionals, and specialist care professionals in obesity treatment. Key elements include: [1] tools that support patients and health professionals in selecting and following up treatment plans [2], scheduling tools that allow primary care professionals to coordinate specialist consultations based on patient needs, and [3] communication platforms that enable secure interactions between patients and healthcare professionals. The figure emphasizes the flow of information between the patient and healthcare providers at the primary healthcare location and between primary and specialist care settings at the hospital. These digital solutions aim to enhance accessibility, ensure continuity of care, and support coordinated decision-making while maintaining relational continuity between patients and providers

Semi-structured interview guides (Supplementary File 1 and 2) were developed collaboratively by the authors, piloted, and revised. The guides included topics such as knowledge and gaps in obesity care, strategies to improve collaboration, and the potential of digital support tools. Questions in the guides were informed by the concept of patient-centered communication, defined as an approach in which healthcare professionals elicit and understand patients’ perspectives, consider their psychosocial context, and engage in shared decision-making aligned with patient values [24].This ensured that topics such as involvement in care, respect for patient preferences, and responsiveness to individual needs were systematically addressed. Both patients and health professionals were shown an illustration of digital solutions to stimulate discussion (Fig. 1).

For healthcare professionals, thematic saturation, defined as the absence of substantively new themes or perspectives across the dataset, was reached after two focus‑group interviews. Decisions were based on repetition of themes, code stability, and sufficient analytic depth relative to the study aim, consistent with established guidance on assessing saturation in qualitative research [25]. For the patient interviews, saturation was monitored iteratively during data collection and analysis. After the tenth interview, no new codes, subthemes, or conceptual insights emerged; two additional interviews confirmed informational redundancy and code stability.

This study was guided by a phenomenological-epistemological approach, focusing on the lived experiences of patients and healthcare professionals and their perspectives on collaboration and digital care. A reflexive thematic analysis (Braun & Clarke, 2006) was selected as it aligns with this orientation and facilitates examination of both shared and divergent experiences across participant groups [26]. All interviews were transcribed verbatim and anonymized. All participants were offered the opportunity to review their transcripts for verification; however, none chose to do so. Data from focus groups and individual interviews were first analysed separately and later integrated. In phase 1, the second author (MWE) transcribed and anonymized the individual interviews, while the first author (ISF) did the same for the focus groups. Patient interviews were coded using NVivo 14 (QSR International NVivo), while focus group data were coded manually with Miro (Miro, Inc.) used as a supportive tool to organize and sort themes in a mind map format. Initial codes were independently generated by ISF (focus groups) and MWE (patients). Any discrepancies in coding were resolved through regular research team discussions. Divergent or negative cases were actively considered, and data not fitting initial themes were discussed to ensure balanced interpretation. Reflexivity was addressed through repeated team discussions, reflecting on how researchers’ assumptions and perspectives could influence coding and interpretation. In phase 2, coding was conducted independently (ISF for focus groups, MWE for individual interviews). Notes taken during transcription informed the coding process to ensure that both perspectives were represented. In phase 3, preliminary themes were identified within each dataset, reflecting both individual patient experiences and systemic challenges in treatment coordination. In phase 4, themes from the two datasets were compared to identify commonalities and discrepancies. Overlapping themes were merged, while unique insights were preserved. In phase 5, themes were refined and structured to highlight central aspects of obesity treatment, including patient–professional relationships, competence in obesity care, and digital collaboration. In phase 6, insights from focus groups and interviews were synthesized to provide a comprehensive understanding of obesity treatment, balancing professional perspectives with patients’ experiences. The first author (ISF) finalized the synthesis to ensure coherence between the two datasets. Key quotes were translated into English for reporting. The COREQ 32-item checklist [27] was completed to enhance transparency.

## Results

Four main themes emerged from the analysis: (1) Communication, trust, and experiences of stigma in patient–professional interactions; (2) Knowledge gaps and coordination across primary and specialist healthcare; (3) Interdisciplinary collaboration in obesity care pathways; and (4) Experiences with digital solutions and in-person care in obesity treatment.

### Communication, trust, and experiences of stigma in patient–professional interactions

Both health professionals and patients emphasized the importance of communication and the patient-professional relationship in obesity treatment. Patients expressed varied preferences regarding how obesity should be addressed by health professionals. While some preferred direct communications, others found a more gradual and sensitive approach to be essential. A common concern among patients was the stigma associated with obesity, which negatively influenced their healthcare experiences. Several patients recounted instances where they felt dismissed, with their symptoms attributed solely to their weight, leading to frustration and reluctance to seek further medical support. Patients described access to more consistent care within the local community as important and associated it with feelings of continuity and being acknowledged beyond weight-related concerns (see quotes Table 3).

Conversely, when health professionals demonstrated understanding and respect, patients reported feeling more comfortable discussing weight-related concerns. Patients described feeling more comfortable and open in consultations when health professionals demonstrated understanding and respect. Health professionals emphasized the importance of a strong therapeutic relationship and described trust as central to patient engagement in obesity treatment.

### Knowledge gaps and coordination across primary and specialist healthcare

Both groups highlighted a need for improved knowledge and structured treatment pathways in obesity care. Patients frequently described encountering primary care professionals with limited expertise in obesity management, noting a reliance on general advice rather than individualized treatment plans. Some patients expressed frustration over the perceived lack of interdisciplinary collaboration and awareness of current treatment options (see quotes Table 3).

Health professionals similarly pointed to inconsistencies in treatment approaches, particularly regarding the timing and appropriateness of different interventions, such as lifestyle changes, medication, and bariatric surgery. While specialists in obesity treatment follow structured protocols, primary care professionals face challenges in determining the right course of action for patients who do not meet referral criteria for specialized services. Several primary care professionals expressed concerns about long waiting times, which often led to a loss of motivation among patients.

Specialists also highlighted difficulties in managing complex obesity cases, particularly when referrals lacked sufficient patient history. Patients described that their medical history and personal progress were not always communicated or understood when transitioning between primary and specialist healthcare. Health professionals also referred to challenges related to information transfer and coordination across healthcare levels.

### Interdisciplinary collaboration in obesity care pathways

Both health professionals and patients emphasized the need for stronger collaboration between primary and specialized healthcare services to ensure continuity of care. Many patients reported falling into a gap between these levels, particularly those who did not meet the strict criteria for specialist care but still required structured treatment (see quotes in Table 3).

Health professionals in both primary and specialist care acknowledge the challenges of coordinating care, particularly for complex cases requiring multidisciplinary input. Primary care professionals noted a lack of clear guidelines for referring patients, whereas specialists highlighted difficulties in obtaining comprehensive patient histories. Both groups called for improved communication and integrated treatment pathways to enhance patient outcomes. Additionally, professionals in specialist care reported difficulties in maintaining patient engagement following discharge from their services. They highlighted a need for structured follow-up in primary care to support long-term weight management. However, primary care professionals expressed concerns about limited resources and insufficient expertise to provide ongoing obesity care (see quotes in Table 3). Both health professionals and patients agreed that interdisciplinary collaborations, such as joint consultations and shared decision-making, could enhance the effectiveness of obesity treatment. Similarly, there was a broad consensus that clearer referral pathways and more clearly defined roles between primary and specialist care could support improved long-term outcomes. Notably, no concerns were raised about treating patients without an existing therapeutic relationship, indicating that structural coordination was regarded as more critical than relational continuity in this context.

### Experiences with digital solutions and in-person care in obesity treatment

Both health professionals and patients recognize the potential benefits of digital solutions in facilitating obesity treatment. Patients viewed digital solutions to improve access to care, particularly for those facing long travel distances or scheduling difficulties. Health professionals noted that digital platforms could enhance interdisciplinary collaboration, streamline patient follow-up, and improve treatment adherence. However, both groups underscored the importance of maintaining personal interactions, particularly during the initial phase of treatment. Patients expressed concerns that digital solutions might reduce the depth of their interactions with health professionals, making it easier to avoid discussing sensitive weight-related issues. Health professionals echo this sentiment, emphasizing that nonverbal cues, emotional engagement, and in-person assessments are crucial, especially for complex cases. A hybrid approach, in which an initial in-person meeting is followed by digital follow-ups, was viewed as a viable model for balancing accessibility and relational care. While some healthcare professionals expressed scepticisms regarding the model’s ability to maintain therapeutic quality over time, patients tended to be more receptive, highlighting its potential to reduce barriers and support continuity in follow-up care (see quotes in Table 3).

**Table 3:** Illustrative quotes from patients and health professionals by theme

Theme	Quote
1. Communication, trust, and experiences of stigma in patient–professional interactions	*“Being met with negative attitudes at the hospital*,* especially when you’re nervous about meeting a new professional*,* truly undermines trust. They act superiorly*,* and you lose faith in them.”* Patient, 45–60 years
	*“I feel more open when the professional shows understanding and listens carefully. It makes discussing my weight concerns easier.”* Patient 30–45 years
	*“Acknowledging patients’ feelings and addressing stigma explicitly helps engagement and adherence in treatment.”* Specialist physician, 15 years’ experience
	*“When they only focus on my weight*,* I feel reduced to a number rather than as a person.”* Patient, 50–65 years
2. Knowledge gaps and coordination across primary and specialist healthcare	*“You must be truly persistent to get help. There’s no clear pathway*,* and it depends on whether the doctor knows what’s available.”* Patient, 30–45 years
	*“Sometimes I feel lost between the hospital and my local clinic. No one seems to have the full picture.”* Patient, 50–65 years
	*“If a patient must wait six months or longer after their referral*,* they often lose motivation*,* and by then*,* the window for effective intervention might have closed.”* Primary care physician, 1.5 years’ experience
	*“Rarely we know what has been done at the specialist level*,* which makes follow-up in primary care challenging.”* Primary care nurse, 8 years’ experience
3. Interdisciplinary collaboration in obesity care pathways	“*Maybe when they ended treatment at the hospital for me*,* they could have referred me to a Healthy Life Centre. I had to do it all on my own.”* Patient 45–60 years
	*“Sometimes I feel like I’m bouncing between different professionals without anyone coordinating my care.”* Patient 35–50 years
	*“Joint consultations and shared decision-making with specialists improve patient outcomes and make care more coordinated.”* Primary care physiotherapist, 5 years’ experience
	*“Without clear roles and shared plans*,* collaboration easily becomes fragmented*,* even when everyone wants to do the best for the patient.”* Specialist healthcare professional, 12 years
4. Experiences with digital solutions and in-person care in obesity treatment	*“You lose something when it’s all digital. It’s harder to read between the lines or pick up on the emotional aspect”.* Patient 30–45 years
	*“Digital follow-ups are convenient*,* but I still need face-to-face interactions for motivation.”* Patient (Male, 40–55)
	*“For many*,* digital follow-ups improve attendance*,* but complex cases require in-person sessions to ensure adherence.”* Specialist, 20 years’ experience
	*“Digital solutions work best when they are built on an existing relationship; otherwise*,* important nuances can be missed.”* Primary care physician, 10 years’ experience

## Discussion

This study explored how health professionals and patients perceive collaboration and the role of digital solutions in improving patient-centered obesity treatment across primary and specialist healthcare in Norway. Our findings address the knowledge gaps, demonstrating how structured collaboration and digital solutions may facilitate coordination across care levels. The findings underscore four interrelated themes- trust and stigma reduction, knowledge gaps and care coordination, integrating digital solutions with in-person care, and interdisciplinary collaboration, as key factors for effective, patient-centered obesity treatment.

### Patient-professional relationships and stigma

Patients emphasized the importance of consistent and respectful communication, which helped reduce misunderstandings and fostered trust. Conversely, encounters characterized by limited obesity-related knowledge or judgmental attitudes negatively affected treatment experiences. This aligns with prior research showing that non-verbal cues and continuity are especially valuable when discussing weight, while limited professional expertise can create barriers [28]. Most patients favour a direct approach to weight management, provided it is delivered respectful [29]. Our findings highlight that enhancing professional competence in obesity care is critical, not only for treatment quality but also for reducing weight stigma. Training that integrates patient-centred communication, awareness of obesity as a chronic condition, and strategies to address sensitive emotions such as shame may help strengthen the therapeutic alliance [30]. Consistent with previous studies, acknowledging and addressing difficult emotions during consultations can mitigate perceived stigma and improve patient trust [18, 19]. Another study pointed out need for patient-centred communication in education and professional training to reduce stigma and improve treatment outcomes [31]. Enhanced education and training about obesity’s complexities could lead to a better understanding of among health professionals, that again can reduce stigma and improve patient treatment experience [32]. Enhancing patients’ experiences of obesity care requires respectful communication, professional expertise, and strategies to reduce stigma. Embedding patient-centered approaches in training and clinical practice may minimize negative experiences, strengthen trust, and promote coordinated care across primary and specialist healthcare.

### Opportunities and challenges of digital solutions in obesity care

Both health professionals and patients viewed digital solutions as useful for improving access, follow-up, information flow, and coordination across care levels. At the same time, they stressed that digital contact should complement rather than replace face-to-face encounters, particularly for sensitive discussions and complex needs.

Despite this shared recognition of potential benefits, our findings reveal a clear contrast between patients’ and healthcare professionals’ perspectives on digital solutions. Patients generally expressed openness and enthusiasm toward digital follow-up, emphasizing improved accessibility, flexibility, and reduced practical barriers to care. For many, digital solutions were perceived as supportive and enabling, particularly when integrated into an established therapeutic relationship. In contrast, healthcare professionals tended to adopt a more cautious stance. While acknowledging the value of digital solutions, they emphasized concerns related to workflow integration, data security, clinical responsibility, and the risk of reduced relational depth over time. Professionals were particularly attentive to how digital formats might limit the interpretation of non-verbal cues and emotional expressions that are central to motivation, adherence, and stigma-sensitive obesity care. Previous studies show that digital solutions can lower logistical barriers, support chronic care follow-up [33] and in some cases achieve weight outcomes comparable to in-person consultations [34]. Structured digital platforms, integrated into existing referral pathways, could therefore enhance coordination while ensuring in-person support when needed.

The importance of continuous, long-term follow-up in obesity care is well documented [35, 36], yet challenges such as technological literacy, data security, and reduced interpersonal engagement remain [37]. Emerging tools, including AI-based solutions, show promise but require validation in clinical practice [38]. While integration into routine care is still limited, digital follow-up and coordination systems may streamline treatment pathways, optimize resource use, and support gradual transition to self-management [39]. These contrasting perspectives underscore the complexity of implementing collaborative digital care in obesity treatment. Patients’ readiness to adopt digital solutions reflects unmet needs related to access, continuity, and flexibility, whereas professionals’ caution highlights structural, ethical, and organizational responsibilities within the healthcare system. Digital solutions therefore cannot be understood as purely technical interventions but must be embedded within existing care pathways and aligned with professional standards, resource availability, and relational care practices.

At the same time, our findings underscore the importance of structured collaboration between primary and specialist care. Importantly, the divergence between patients’ and professionals’ perspectives also reflects their different positions within the healthcare system. Patients primarily evaluated digital solutions based on lived experiences of access and continuity, whereas healthcare professionals assessed them through the lens of clinical accountability, coordination demands, and system-level constraints. Recognizing and addressing these differing priorities is essential for developing digital solutions that support both patient-centeredness and sustainable interprofessional collaboration. Patients in our study valued the accessibility of primary care but often sought specialist expertise for complex challenges, while professionals stressed the need for clear pathways and interdisciplinary teamwork. Digital solutions may enable specialists to take an advisory role, ensuring timely support while preserving the continuity of primary care relationships. Digital solutions may facilitate this by allowing specialists to adopt a more advisory role, ensuring timely support while preserving the relational continuity of primary care. Strengthening patient-centeredness in obesity care thus requires both interpersonal trust and system-level improvements, with digital solutions offering opportunities to bridge gaps when implemented as a complement to, rather than a replacement for, in-person interactions. Taken together, the findings indicate a structural tension between relationship-based continuity in primary care and the more specialized approach in hospital-based obesity treatment. Fragmented information flow across healthcare levels may hinder coordinated care and timely interventions. Strengthening communication structures and collaboration between care levels therefore appears essential for improving patient-centered obesity care. Future studies should include a broader and more diverse sample of patients and healthcare professionals to capture varying perspectives. Longitudinal and implementation-focused research could further explore how digital solutions and collaborative care models are adopted and impact patient-centered obesity treatment across healthcare levels. These insights would inform both practice and policy.

### Strengths and limitations

This study combined focus group interviews with health professionals and individual interviews with patients, allowing for data triangulation and multiple perspectives, which strengthens credibility [40]. However, it also introduces differences in the type of data generated, as focus groups capture group dynamics while individual interviews provide more in-depth personal reflections [41]. As the interview forms are not directly comparable, this may have influenced the scope and nature of the insights obtained. However, this approach was intentional, aiming to capture complementary perspectives from both patients and health professionals and to provide a rich, integrated understanding of communication, collaboration, and digital solutions in obesity care.

The health professional sample was recruited through snowball sampling, which may have introduced bias toward informants with particular interest or experience in digital solutions and collaborative care. Furthermore, the absence of certain health professional groups in the focus groups, such as psychologists, may restrict insights into psychosocial care aspects. Additionally, the patient sample was relatively small (*n* = 12) and predominantly female (10 of 12 participants). This gender distribution may have influenced the findings, particularly in relation to experiences of stigma, openness to digital solutions, and perceptions of collaboration with healthcare professionals. However, the predominance of female participants reflects the clinical reality of obesity treatment, in which women constitute the majority of patients seeking and receiving care [42]. Although male perspectives may therefore be underrepresented, the sample is broadly representative of gender patterns within obesity care.

All interviews were conducted digitally via Teams. While this facilitated participation and reportedly increased comfort and openness among patients, it may have constrained the observation of non-verbal cues and subtle interpersonal dynamics. Participant familiarity with digital solutions varied potentially shaping perceptions. Finally, while data were transcribed and translated into English, some nuances may have been lost in translation.

## Conclusion

Our findings highlight the need to balance relational, structural, and technological dimensions in obesity care. Trust and respectful, patient-centered communication is fundamental for engagement and long-term adherence, yet effective collaboration between primary and specialist services remains a persistent structural challenge. While patients generally expressed openness toward digital solutions to enhance accessibility and continuity, healthcare professionals emphasized the need for cautious integration to safeguard clinical quality, relational depth, and system-level responsibilities. Digital solutions therefore hold promise for improving coordination and follow-up, but only when embedded within care pathways that preserve relational continuity and clarify professional roles. By strengthening both human and technological dimensions, obesity care may become more sustainable and patient-centered, with important implications for clinical practice, health policy, and future service development.

## Supplementary Information

Below is the link to the electronic supplementary material.

Supplementary Material 1

Supplementary Material 2

## Data Availability

The datasets generated and analysed during the current study are not publicly available due to the small number of informants, and the risk of identification. Only anonymized and aggregated findings are included in this article. Data may be available from the corresponding author on reasonable request.
