# Supplementary material for: Obesity treatment across healthcare levels: collaboration and digital solutions - a qualitative study of health professional and patient perspectives
Source: BMC Health Serv Res. 2026 Feb 6;26:341. doi: 10.1186/s12913-026-14155-4 (PMC12973644; doi:10.1186/s12913-026-14155-4)
Supplement: Supplementary file 1 — Supplementary Material 1 [file 12913_2026_14155_MOESM1_ESM.pdf]

Supplementary File 1

Focus Group Interview Guide for the study:

Obesity treatment across healthcare levels: collaboration and digital solutions

Number of informants in the focus groups: 5–7 persons

One interviewer will lead the interview and ask questions, while the other will be a moderator, monitoring time and taking notes.

Tools used: Digital recorder and visualized idea (**Figure 1)**.

About the interview:

The focus group interviews followed a semi-structured format, addressing three main topics:

1. Knowledge and gaps in obesity care
2. Strategies to improve collaboration and resource allocation
3. The potential of digital support tools to enhance treatment outcomes and optimize services

The interview guide ensured that the same topics were covered while leaving room for participants to raise their own perspectives. To stimulate discussion, informants were shown an illustration (**Figure 1**) depicting how digital solutions could facilitate communication and collaborative care in obesity treatment.

Focus group interview structure

Time	Topic	Main Questions	Follow-up Prompts
**15 min**	Background	Please introduce yourself:<br>- Age<br>- Current role and specialization	–How long have you worked in healthcare?<br>- How long have you worked with patients with obesity?
**15 min**	1. Knowledge and gaps in obesity care	- How do you view the general level of knowledge in the obesity field?	Has knowledge changed since you started working in this area?<br>- What is the impact of limited knowledge in practice?
**20 min**	2. Collaboration and resource allocation	-What do you think is important for achieving better collaboration across healthcare levels for patients with obesity?	- Patient story: Can you think of a patient with obesity where collaboration did not work well?<br>- What were the reasons, and what consequences did this have for the patient?<br>- Involvement of several providers<br>- Organizational factors
**20 min**	3. Digital support tools	- What do you think is needed to improve obesity treatment in the future?<br>- How do you envision the ideal treatment?<br>- Do you have ideas for new solutions or tools that could improve treatment?	- Patient–provider relationship<br>- Inter-professional collaboration<br>- Barriers and facilitators
**20 min**	Discussion of illustration (**Figure 1**)	We would like your feedback on the presented illustration of digital tools to support communication and coordination in obesity care across healthcare levels. Examples include scheduling tools, communication platforms, and decision-support tools for GPs.	- What is important for you/others to start using and to benefit from these solutions?
**10min**	Closing	At the end of the interview:<br>- Are there other important aspects we have not discussed that you would like to mention?<br>- Is there anything else you would like to add related to what we talked about today?	- Important for you?<br>- Important for your patients?
