# Supplementary material for: Obesity treatment across healthcare levels: collaboration and digital solutions - a qualitative study of health professional and patient perspectives
Source: BMC Health Serv Res. 2026 Feb 6;26:341. doi: 10.1186/s12913-026-14155-4 (PMC12973644; doi:10.1186/s12913-026-14155-4)
Supplement: Supplementary file 2 — Supplementary Material 2 [file 12913_2026_14155_MOESM2_ESM.pdf]

Semi-Structured Interview Guide for Patients

Opening Questions

- To begin, could you tell me a bit about yourself?
  - Your **age**
  - How long you have been in **obesity treatment**?
  - Your approximate **BMI?**
- Is there anything about your background that you feel is important for me to understand before we talk about your treatment experiences?

1. Treatment Experiences and Perceptions of Expertise

- Can you describe your experiences with obesity treatment so far?
- What types of treatment or services have you received (e.g., lifestyle programs, digital tools, specialist services, primary care follow-up)?
- How would you describe the professional expertise you have encountered during treatment?
- Have you felt that the health professionals involved had the necessary knowledge to support your treatment needs?

Optional probes:

- Differences between primary and specialist care
- Consistency or variability in advice received

2. The Patient–Professional Relationship

- How would you describe your relationship with the different health professionals involved in your care?
- What aspects of these relationships have been most important to you?
- How has your primary care physician (fastlege) contributed to your treatment journey?
- Have there been moments where communication or support from health professionals made a big difference, positive or negative?

Optional probes:

- Trust, safety, openness
- Experiences of being heard or not heard

3. Competence and Attitudes Encountered in Healthcare

- How would you describe the attitudes or views you've encountered from health professionals regarding obesity?
- Have you experienced understanding, support or empathy in your treatment?
- On the other hand, have you encountered attitudes that felt unhelpful, stigmatizing or discouraging?
- What impact have these experiences had on your motivation or treatment journey?

Optional probes:

- Avoid naming individuals; focus on experiences
- Differences across healthcare levels

4. Experiences with Digital Solutions and Tools

- Have you used any digital tools as part of your obesity treatment (e.g., video consultations, apps, remote monitoring)?
- How did you experience using these digital solutions compared to in-person care?
- What do you think about the amount of time available during digital vs. in-person consultations?
- Do you believe digital tools could help improve communication or collaboration among health professionals involved in your care?
- In your opinion, what works well digitally—and what still works best face-to-face?

Optional probes:

- Ease of use
- Personalization
- Feeling supported or not supported digitally

5. Closing Questions

- Is there anything we have not discussed that you feel is important for understanding your experiences with obesity treatment?
- Do you have any final reflections on how digital and in-person care could better support collaboration and patient-centred treatment?
